# Performance of four equine pain scales and their association to movement asymmetry in horses with induced orthopedic pain

**DOI:** 10.3389/fvets.2022.938022

**Published:** 2022-08-12

**Authors:** Katrina Ask, Pia Haubro Andersen, Lena-Mari Tamminen, Marie Rhodin, Elin Hernlund

**Affiliations:** ^1^Department of Anatomy, Physiology and Biochemistry, Swedish University of Agricultural Sciences, Uppsala, Sweden; ^2^Department of Clinical Sciences, Swedish University of Agricultural Sciences, Uppsala, Sweden

**Keywords:** pain assessment, lameness, LPS induction, objective gait analysis, movement symmetry, reliability

## Abstract

**Objective:**

This study investigated the relationship between orthopedic pain experienced at rest, and degree of movement asymmetry during trot in horses with induced reversible acute arthritis. Orthopedic pain was assessed with the Horse Grimace Scale (HGS), the Equine Utrecht University Scale of Facial Assessment of Pain (EQUUS-FAP), the Equine Pain Scale (EPS), and the Composite Orthopedic Pain Scale (CPS). Reliability and diagnostic accuracy were evaluated with intraclass correlation coefficients (ICC) and area under the curve (AUC).

**Study design and animals:**

Eight healthy horses were included in this experimental study, with each horse acting as its own control.

**Methods:**

Orthopedic pain was induced by intra-articular lipopolysaccharide (LPS) administration. Serial pain assessments were performed before induction and during pain progression and regression, where three observers independently and simultaneously assessed pain at rest with the four scales. Movement asymmetry was measured once before induction and a minimum of four times after induction, using objective gait analysis.

**Results:**

On average 6.6 (standard deviation 1.2) objective gait analyses and 12.1 (2.4) pain assessments were performed per horse. The ICC for each scale was 0.75 (CPS), 0.65 (EPS), 0.52 (HGS), and 0.43 (EQUUS-FAP). Total pain scores of all scales were significantly associated with an increase in movement asymmetry (*R*^2^ values ranging from −0.0649 to 0.493); with CPS pain scores being most closely associated with movement asymmetry. AUC varied between scales and observers, and CPS was the only scale where all observers had a good diagnostic accuracy (AUC > 0.72).

**Conclusions and clinical relevance:**

This study identified significant associations between pain experienced at rest and degree of movement asymmetry for all scales. Pain scores obtained using CPS were most closely associated with movement asymmetry. CPS was also the most accurate and reliable pain scale. All scales had varying linear and non-linear relations between total pain scores and movement asymmetry, illustrating challenges with orthopedic pain assessment during rest in subtly lame horses since movement asymmetry needs to be rather high before total pain score increase.

## Introduction

Painful pathology in the locomotor apparatus often leads to increased movement asymmetry, due to decreased loading of the painful limb, i.e., lameness. Nonetheless, horses perceived as sound by their owners commonly show movement asymmetry (1, 2), and it remains unclear how the degree of movement asymmetry is associated with the level of pain experienced. Changes in behavior and in facial expressions have been recognized and assessed with an ethogram in ridden horses with clinical orthopedic pain (3, 4), but have not yet been associated to different degrees of movement asymmetry detected by objective gait analysis.

Different types of orthopedic pain during rest, including moderate and severe post-surgical orthopedic pain (5), laminitis (6), and induced inflammatory arthritis (7), have been successfully assessed using different pain assessment tools. We recently showed that a number of body behaviors and facial expressions included in those tools predict mild orthopedic pain in resting horses (8). However, it is not known whether these pain assessment tools can recognize resting pain displays associated with movement asymmetry in a reliable and accurate way. A clinically relevant question in that regard is whether increased pain score and movement asymmetry occur simultaneously or not.

In addition, different pain pathologies may generate different pain displays (9) and a pain assessment tool may therefore only be valid for the pain types specified in the validation study. Pain *per se* is associated with a number of general features, but the anatomical location of the pain will induce different compensatory body behaviors, such as increased movement asymmetry due to decreased weight bearing during orthopedic pain. Facial displays of pain, on the other hand, are thought to be general for acute pain or acute exacerbations of chronic pain in most mammals, including horses (10). Indeed, grimace-based pain scales developed for horses experiencing post-surgical castration pain (11) and acute visceral pain (12) seem to identify laminitis (6), post-surgical orthopedic pain (5), and head-related pain (13) successfully. Whether a behavior- or grimace-based pain scale performs better on the same type of orthopedic pain has not been evaluated, but assessment of behaviors and facial expressions together has been recommended to optimize pain detection (14, 15).

Understanding the relationship between pain experienced at rest and degree of movement asymmetry during motion can aid the investigation of whether a movement asymmetry is caused by pain or not. Adding a pain assessment tool during rest to the lameness examination may thus be helpful in deciding the pain level in the equine orthopedic patient. For this use, proper validation of the pain assessment tool is essential, since validation and high observer reliability in experimental settings do not necessarily mean that a pain assessment tool performs well under clinical conditions (16). For instance, observers are commonly trained prior to pain assessment to improve reliability in experimental studies, while observer training may not be possible under clinical conditions, especially with the current lack of standardized training protocols and purpose-made teaching material. Blinding of observers to the animal's pain status in experiments is also common, but in a clinical setting the clinician very often has information or beliefs about the pain status of the patient, for example knowing the diagnosis or treatment, and thereby if the horse is lame or in post-surgical pain.

This study therefore had two aims: (1) to investigate the relationship between orthopedic pain experienced at rest and degree of movement asymmetry during trot in horses; and (2) to compare, under clinical conditions, the performance parameters of pain assessment tools containing varying categories of facial expressions and body behaviors.

Four existing pain assessment tools were applied simultaneously by three observers immediately before and after serial objective measurements of movement asymmetry ranging from baseline conditions to painful conditions, and back to baseline. The hypotheses tested were that increased pain scores are associated with increased movement asymmetry, and that scales containing both body behaviors and facial expressions perform better than scales with only behavioral or facial items. A final hypothesis was that the reliability of the pain assessment tools would be similar to previous published values.

## Material and methods

### Ethical approval

The study was approved by the Swedish Ethics Committee (diary number 5.8.18-09822/2018) in agreement with Swedish legislation on animal experiments. As outlined in EU Directive 2010/63/EU on animal experiments, replacement, reduction, and refinement were carefully considered in the study design. The ARRIVE guidelines were followed (17) and the data collected can be used for multiple purposes.

### Animals and experimental design

The data were collected as part of a previous study (7). In brief, seven healthy Standardbred trotters and one Warmblood horse [mean (standard deviation, SD) age 14.5 (3.7) years, body mass 552 (39) kg, height at withers 160 (2.78) cm] were recruited for the experiment. Exclusion criteria were lameness grade >1, scored during straight line trot on a 0–5 ordinal scale (0 = sound and 5 = non-weight bearing lameness) or any significant clinical findings after a full clinical examination.

An experimental study was conducted with each horse as its own healthy control. Movement asymmetry was measured using objective gait analysis (section Objective gait analysis) on one occasion before induction of lameness (baseline) and a minimum of four times after induction, until each horse had returned to its baseline movement asymmetry. Pain was evaluated in the box stalls using four pain scales, directly before and after each objective gait analysis (section Pain assessment). Baseline measurements were performed after 10–12 days of acclimatization, and acute short-term inflammatory arthritis was induced 1 or 2 days later by administering lipopolysaccharides (LPS) into the tarsocrural joint of the pelvic limb with the highest pre-existing movement asymmetry. A 3 ml solution of LPS from *Escherichia coli* O55:B5 1 mg/ml (*L5418 Sigma*), with a stock concentration of 1.167 ng/ml, was administered to the dorsomedial pouch after evacuation of 3 ml synovia, using routine aseptic techniques.

If the horse was judged to be too lame to trot, corresponding to lameness grade >3/5 on a 0–5 ordinal scale, a protocol for rescue analgesia was initiated. This protocol consisted of evacuation of synovia to decrease joint distension and lessen inflammatory load and pain. Measurements were then continued when the lameness grade decreased.

### Objective gait analysis

Movement asymmetry was measured at walk and trot, on a straight line on hard and soft surfaces and during lunging on a soft surface. For horses with subjectively increased movement asymmetry at the lunge, a second straight-line trot measurement was performed on the hard surface after lunging. During motion, the positions of seven spherical markers (38 mm diameter, Qualisys AB, Sweden) attached to the horse were recorded in 3D at 200 Hz, using 13 infrared optical motion capture cameras (Qualisys AB, Sweden) and tracked by the QTM software (version 2.11-2019.3, Qualisys AB, Sweden). Lameness was subjectively assessed during ongoing measurements by experienced equine veterinarians, one of whom also participated in the pain assessments. Data from the first and, when present, the second straight-line trot on hard surface were used for further analysis. Only the vertical traces from head and pelvic markers were extracted for calculation of lameness metrics, using custom-written scripts in MatLab (18). Details on filtering and stride segmentation can be found elsewhere (19, 20). To cover different strategies used by the horses to decrease loading of the pelvic limb in pain (impact lameness), differences in minimum height between the left and right stance phase of each stride were computed, resulting in HD_min_ for the head marker and PD_min_ for the pelvic marker. These are two variables that change in horses with weight-bearing pelvic limb lameness and with a compensatory head nod (21, 22). Trial means of HD_min_ and PD_min_ were computed and negative left-side means were converted to positive right-side means. To illustrate the change in overall movement asymmetry after induction, a total asymmetry score (TAS) in mm was calculated by adding together absolute differences in HD_min_/2 and PD_min_ from baseline movement asymmetry. Subjective lameness scores were not included in calculation of TAS.

### Pain assessment

Pain was evaluated directly from outside the box stall using the Horse Grimace Scale (HGS) (11), the Equine Utrecht University Scale of Facial Assessment of Pain (EQUUS-FAP) (12), the Equine Pain Scale (EPS) (23), and the Composite Orthopedic Pain Scale (CPS) (7). These scales consist of multiple items assessing facial expressions, behaviors, and/or physiological variables. Item scores are added to give a total pain score ranging from 0 to 12 (HGS), 0 to 18 (EQUUS-FAP), 0 to 30 (EPS), or 0 to 39 (CPS). HGS was originally designed for pain assessment from video or footage, while the other scales are applicable for live assessment. Observation time was 2 min for HGS, EQUUS-FAP, and EPS, and 5 min for CPS.

The same horse was observed by three pain assessors, simultaneously and independently assigning the horse a total pain score with each of the pain scales, always used in the same order (HGS, EQUUS-FAP, EPS, and CPS). This was defined as one pain assessment, and yielded HGS, EQUUS-FAP, EPS, and CPS pain scores from observer 1, from observer 2, and from a third observer. Observers 1 and 2 participated in all assessments, while the third observer was one of observer 3, 4, or 5. All observers, except observer 1 who participated during objective gait analyses, were blinded to limb of induction and lameness grade, and only observed the horses in their box stalls. Observers 1–3 were equine veterinarians, with experience of pain assessment, observer 4 was an agronomist, and observer 5 was an equine ethologist. All had private and/or professional equestrian experience. Prior to the study, the observers familiarized themselves thoroughly with the pain scales, through reading published scientific papers and score sheets/descriptions, but did not train on videos or live horses.

### Statistics

All statistical analyses were conducted in R (24). Descriptive statistics for pain assessment and movement symmetry data were calculated and plotted with “ggplot2” (25). Normality of the dataset was evaluated with Shapiro Wilks test (*p* < 0.05 indicating non-normality) and visually with histograms. Due to non-normality, median and 1st and 3rd interquartile were calculated for total pain scores. Reliability was analyzed with intraclass correlation (ICC) coefficient (26), by computing two-way random ICC_agreement_ (ICC2, A1; “iccNA”). The level of reliability was categorized according to an existing system (27).

To estimate construct validity, the change in TAS was used as a proxy for pain intensity. To enable identification of non-linear associations, the association between total pain score and TAS was tested with generative additive mixed models (“gamm”) (28, 29), with total pain score as dependent variable and TAS as explanatory variable. “Horse” was included as a random effect and an autocorrelation effect was added to handle similarity between observations over time. To enable comparison between horses, the effect of time was standardized by the use of a proportional time scale. The maximum change in TAS was set at 50%, the baseline at 0%, and the last measurement at 100%. Information on whether a pain assessment was performed before or after an objective gait analysis was also included. As the model could not handle crossed random effects, separate models were run for observers 1–5. The explained deviance (*R*^2^ value) of the model for each scale was noted, and residuals were plotted and evaluated visually.

Performance of pain scales were further evaluated with area under the curve (AUC) generated from receiver-operating characteristic (ROC) curves. AUC is a measure of the probability that an observation classified as “pain” is ranked higher than an observation classified as “no pain” – the higher the probability the better accuracy (30). Prediction outcomes were computed from the generative additive mixed models (“predict”) (31) and used as predictive values when computing ROC curves (“roc”) and AUC with 95% confidence intervals (CI) (“auc,” “ci”) (32). The change in TAS defined the pain status of the horse in each observation, hence, TAS > 10 categorized the horse as in pain and TAS ≤ 10 categorized the horse as free from pain. This is a cut-off value, resembling a mild lameness grade. The AUC was classified according to previously described thresholds (33).

## Results

**Figure 1:**
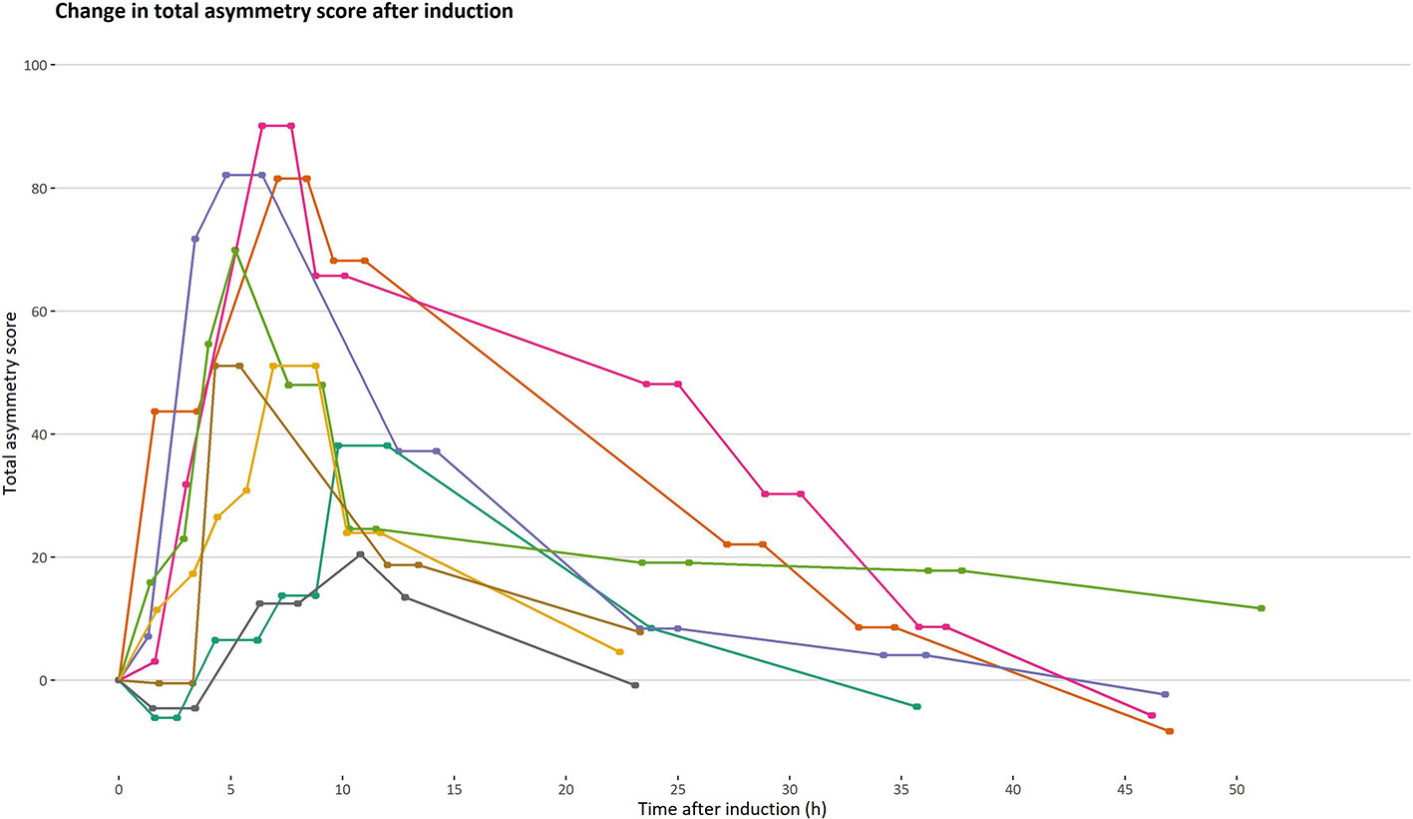
Progression and regression of total asymmetry score after induction of orthopedic pain. Total asymmetry score is presented on the *y*-axis. A timeline is presented on the *x*-axis to illustrate changes in total asymmetry unaffected by time. Each line represents one horse (*n* = 8) and each point on a line represents an occasion where an objective gait analysis was performed.

Lameness was successfully induced in all horses (three right and five left pelvic limbs). Rescue protocol was initiated in two horses, where evacuation of synovia was sufficient to decrease joint distension and lameness grade. All objective gait analyses and pain assessments before and after evacuation of synovia were included in the analysis. In total, 53 measurements of objective gait analysis were performed, with a mean (SD) number of occasions of 6.6 (1.2). Mean (SD) increase in TAS after induction was 27 mm (26). The time points for measurement differed between horses, as did the time with increased TAS, due to individual responses to the induction (Figure 1). All horses returned to baseline movement asymmetry within 52 h after induction. Details of changes in asymmetry over time and absolute values of HD_min_ and PD_min_ are provided in Supplementary Materials (Supplementary File S1).

**Figure 2:**
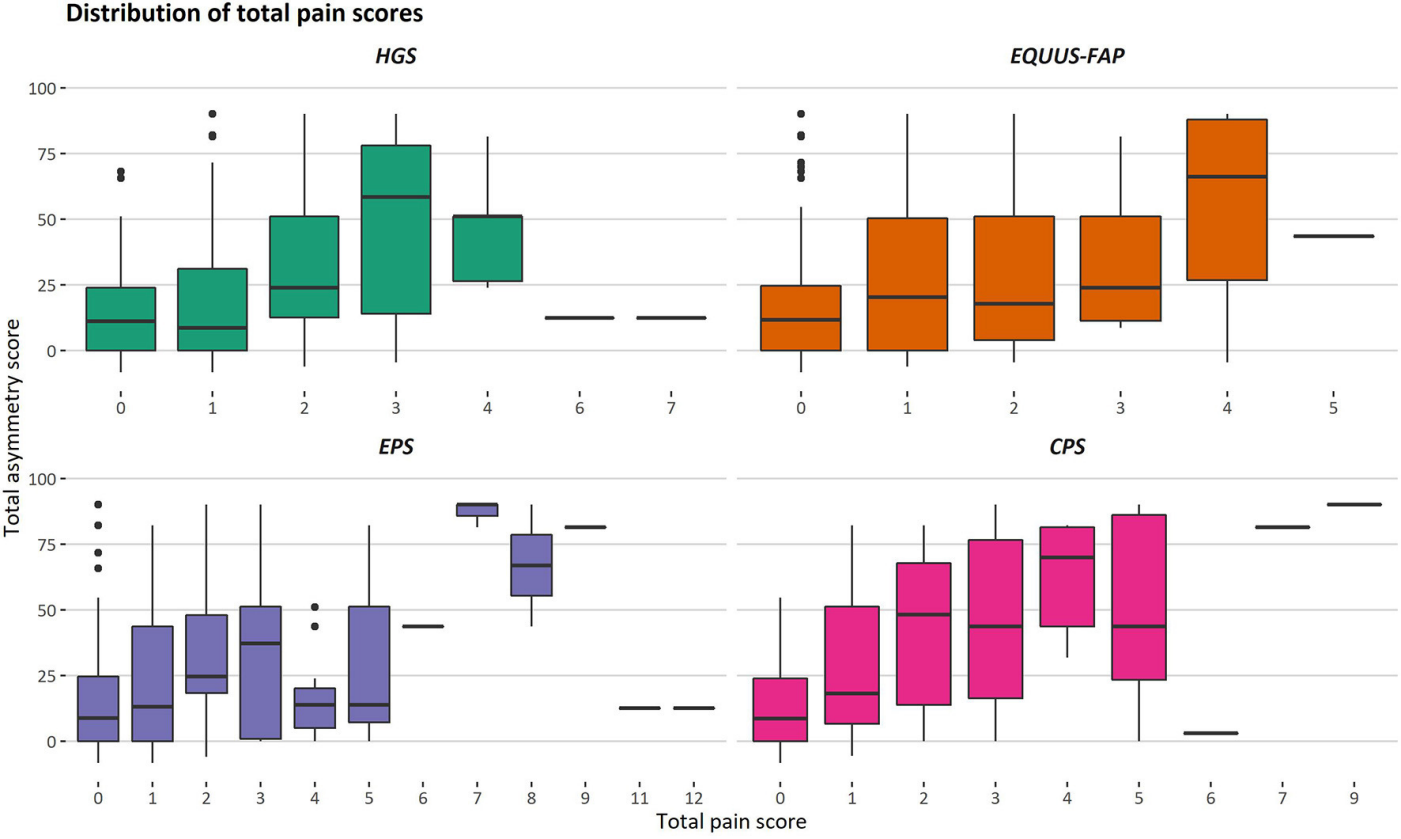
Distribution of total pain scores for different degrees of movement asymmetry. Total asymmetry scores are presented on the *y*-axis, where 0 is the objective gait analysis performed before induction. Total pain scores given with the Horse Grimace Scale (HGS), Equine Utrecht University Scale of Facial Assessment of Pain (EQUUS-FAP), Equine Pain Scale (EPS), and Composite Orthopedic Pain Scale (CPS) are presented on the *x*-axis. Each box illustrates the distribution of one pain score over different degrees of movement asymmetry, given by three observers. The median is presented as the black line in a box, and the lower and upper ends of the boxes show the lower and upper quartile. The lower and upper whiskers show the lowest and highest 25% of the data. Outliers are shown as black dots.

During the study, 97 pain assessments were performed, with a mean (SD) number of 12.1 (2.4) pain assessments per horse. There was considerable variation in total pain scores for both low and high total asymmetry scores (Figure 2), and total pain scores >0 were present for pain assessments before induction for all scales (Table 1). As illustrated in Figure 2 and Table 1, the majority of total pain scores were at the low end of each scale's range. The highest pain score reached 58.3% (HGS), 27.8% (EQUUS-FAP), 40% (EPS), or 23.1% (CPS) of each scale's maximum total pain score.

**Table 1:** Median, 1^st^ and 3^rd^ interquartile (IQ), minimum (min) and maximum (max) total pain score for pain assessments, made before and after pain induction, using the Horse Grimace Scale (HGS), Equine Utrecht University Scale of Facial Assessment of Pain (EQUUS-FAP), Equine Pain Scale (EPS), and Composite Orthopedic Pain Scale (CPS).

**Scale**	**Pre-induction**				**Post-induction**			
	**Median**	**1st−3rd IQ**	**Min**	**Max**	**Median**	**1st−3rd IQ**	**Min**	**Max**
**HGS (0–12)**	1.00	0.00–1.00	0.00	3.00	1.00	0.00–1.00	0.00	7.00
**EQUUS-FAP (0–18)**	0.00	0.00–1.00	0.00	2.00	0.00	0.00–1.00	0.00	5.00
**EPS (0–30)**	0.00	0.00–2.25	0.00	5.00	1.00	0.00–2.00	0.00	12.00
**CPS (0–39)**	0.00	0.00–0.00	0.00	5.00	0.00	0.00–1.00	0.00	9.00

The total pain score range is stated in brackets after each scale.

The only scale with good reliability was CPS, with an ICC coefficient (95% confidence interval, CI) of 0.753 (0.675–0.818). EPS and HGS were both moderately reliable with an ICC coefficient (95% CI) of 0.648 (0.548–0.736) and 0.522 (0.406–0.631), respectively. EQUUS-FAP showed poor reliability, with an ICC coefficient (95% CI) of 0.432 (0.310–0.552).

**Figure 3:**
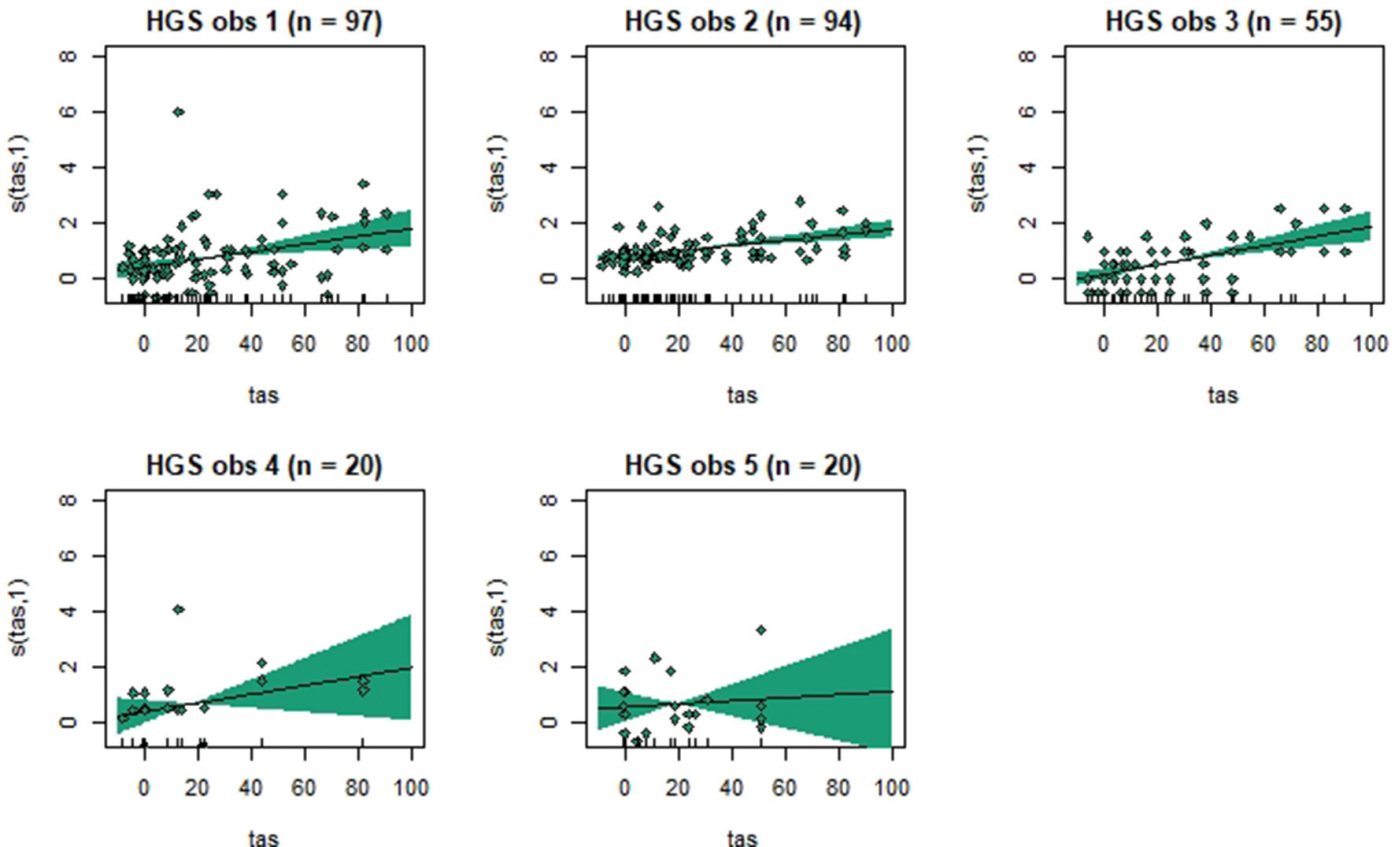
Association between total pain scores from the Horse Grimace Scale (HGS) and total asymmetry scores (TAS), analyzed using generalized additive mixed models. The *y*-axis on the partial effects plots shows total pain scores (maximum total pain score on the scale is 12), with the estimated degrees of freedom (EDF) in brackets. 1 indicates a linear relationship and 3 a cubic function. The *x*-axis shows the total asymmetry score (TAS) in mm, where 0 is the baseline objective gait analysis. Residuals are plotted in the graphs. The shaded areas indicate the 95% confidence intervals. The number of observations made by each observer is shown as *n*.

**Figure 4:**
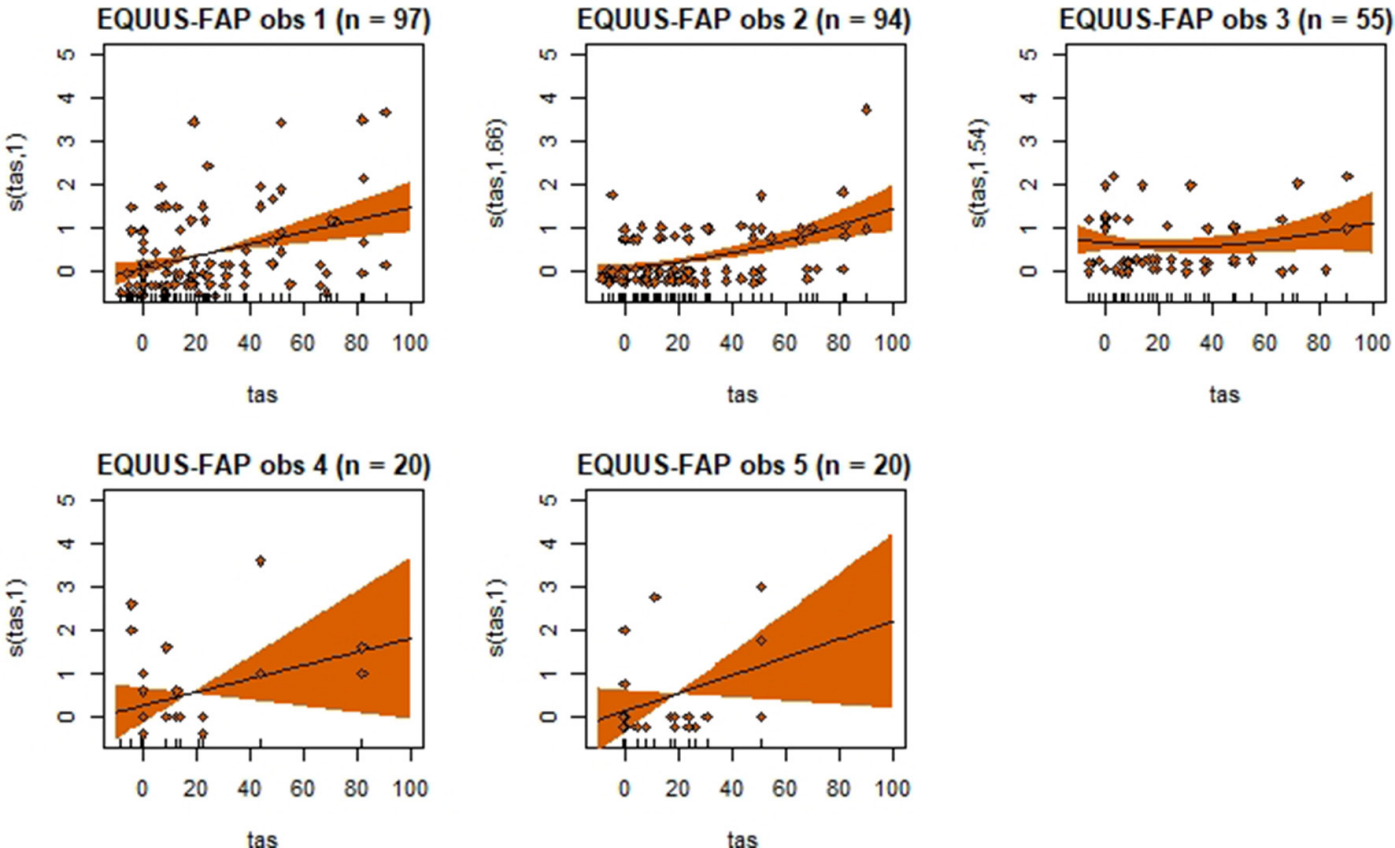
Association between total pain scores from the Equine Utrecht University Scale of Facial Assessment of Pain (EQUUS-FAP[[Inline Image]]) and total asymmetry scores (TAS), analyzed using generalized additive mixed models. See the caption to Figure 3 for more details. Maximum total pain score on the scale is 18.

**Figure 5:**
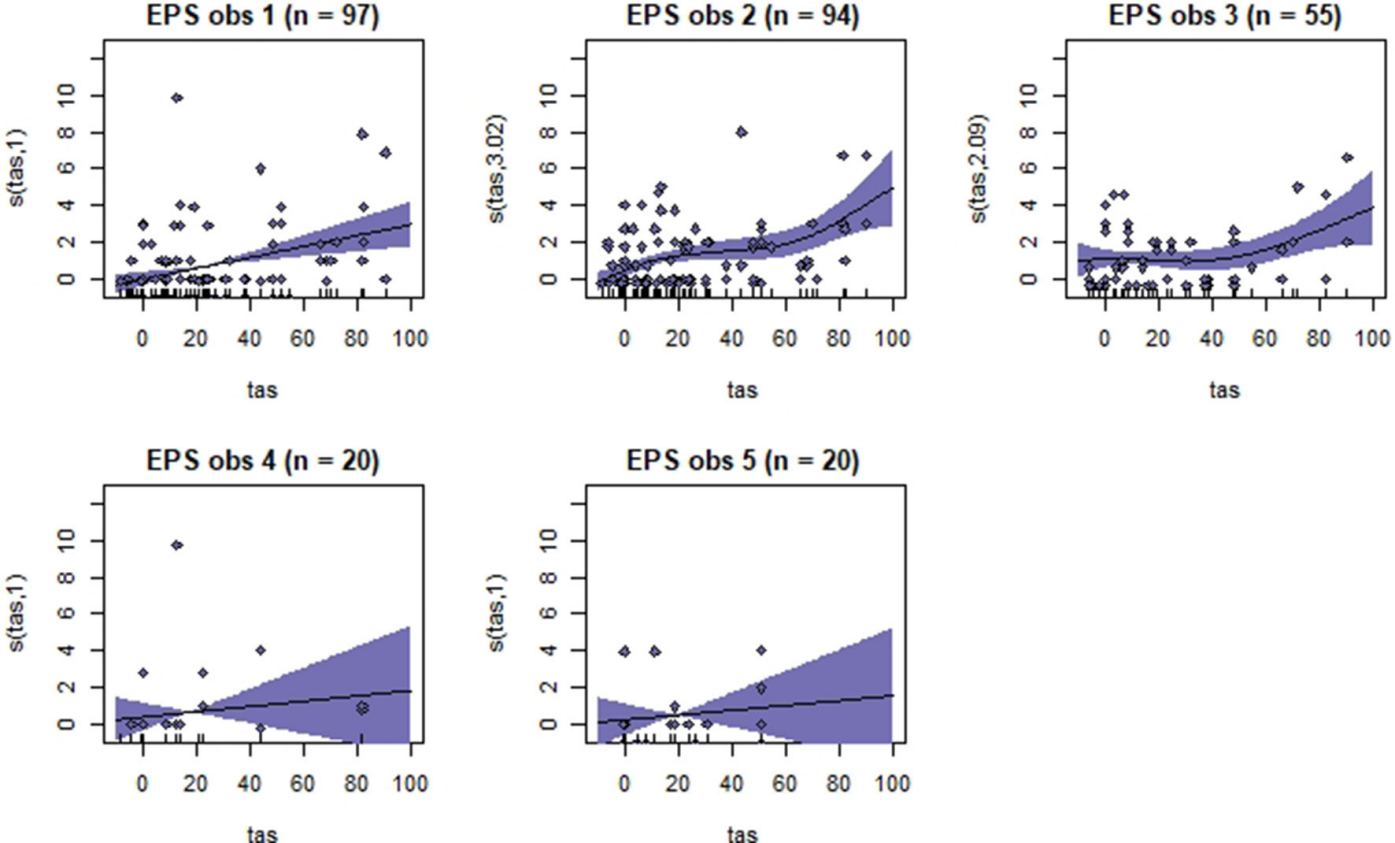
Association between total pain scores from the Equine Pain Scale (EPS) and total asymmetry scores (TAS), analyzed using generalized additive mixed models. See the caption to Figure 3 for more details. Maximum total pain score on the scale is 30.

**Figure 6:**
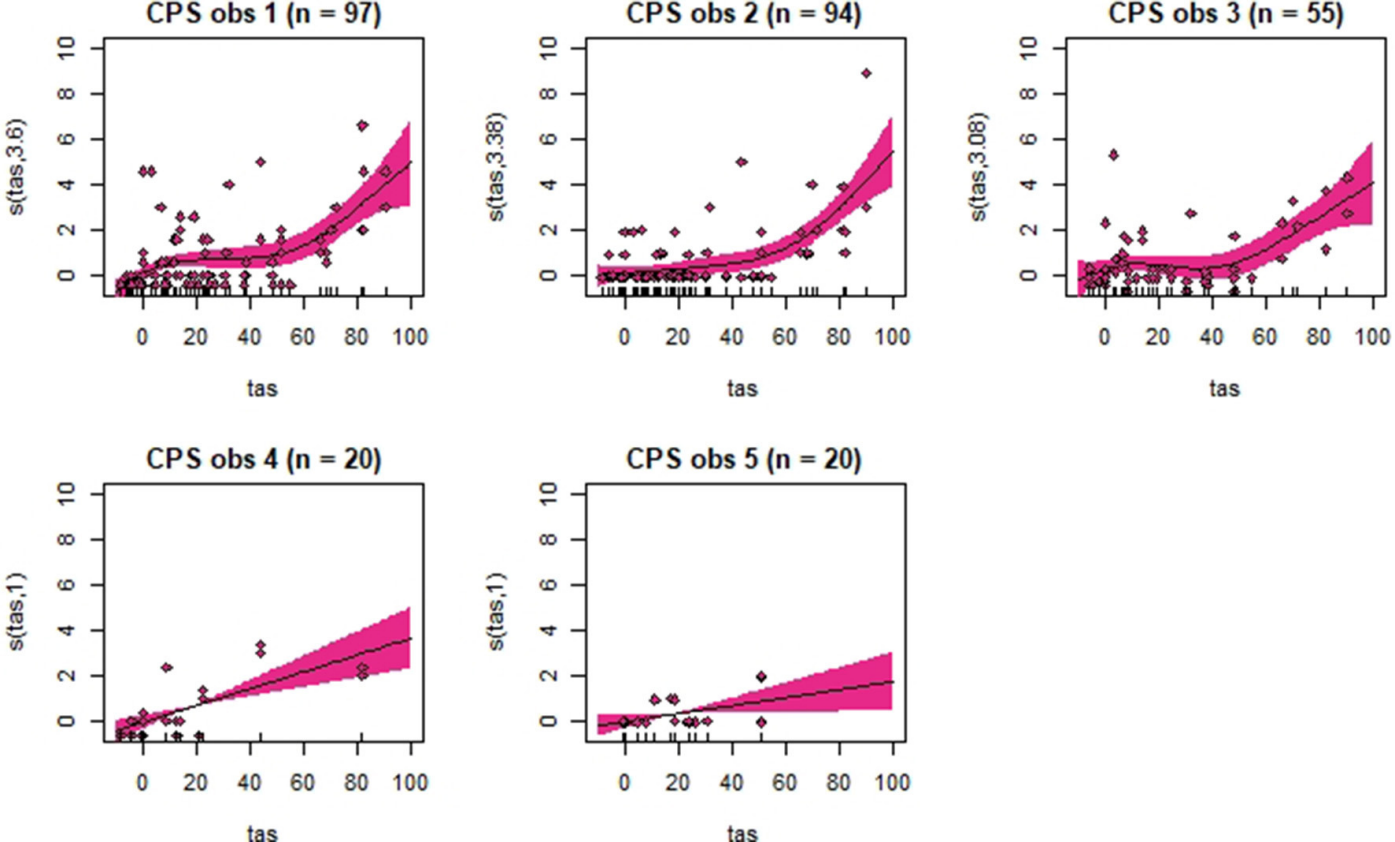
Association between total pain scores from the Composite Orthopedic Pain Scale (CPS) and total asymmetry scores (TAS), analyzed using generalized additive mixed models. See the caption to Figure 3 for more details. Maximum total pain score on the scale is 39.

Generalized additive mixed models revealed significant associations between total pain scores and TAS on a normalized timeline for all scales, but not for all observers (Table 2). CPS had a significant association between total pain scores and TAS for all observers, while the other scales had significant associations for three (EPS and HGS) or two (EQUUS-FAP) observers. The *R*^2^ values ranged from −0.0649 to 0.493, and showed that TAS explained higher variance in total CPS pain scores for most observers compared with pain scores of the other scales. On several occasions, total pain scores before performing objective gait analysis were significantly higher than pain scores given after objective gait analysis (Table 2). HGS had significantly higher pain scores before objective gait analysis for observers 1–4, EQUUS-FAP for observers 1 and 4, and EPS for observer 1. Partial effects plots were created to depict the changing linear and non-linear relationship between total pain scores and total asymmetry scores (Figures 3–6). Visual evaluation of the plots showed that many points did not follow the estimated line and confidence interval, indicating great variance in the data that was not explained by the model. The plots also showed that pain scores above moderate level were rare and not necessarily present when TAS was high in our experimental model.

**Table 2:** Results of generalized additive mixed models for the Horse Grimace Scale (HGS), Equine Utrecht University Scale of Facial Assessment of Pain (EQUUS-FAP), Equine Pain Scale (EPS), and Composite Orthopedic Pain Scale (CPS), where each observer (1–5) is modeled separately.

	**HGS**			**EQUUS-FAP**		
	**Association (*p*-value)**	**Type (*p*-value)**	***R*^2^ value**	**Association (*p*-value)**	**Type (*p*-value)**	***R*^2^ value**
**Observer 1 (*****n*** **=** **97)**	0.00159^**^	0.00127^**^	0.125	<0.001^***^	0.0147^*^	0.13
**Observer 2 (*****n*** **=** **94)**	<0.001^***^	0.0273^*^	0.083	<0.001^***^	0.109	0.214
**Observer 3 (*****n*** **=** **55)**	<0.001^***^	0.019^*^	0.304	0.4	0.292	0.035
**Observer 4 (*****n*** **=** **20)**	0.189	0.0259^*^	0.0884	0.19	0.0267^*^	0.205
**Observer 5 (*****n*** **=** **20)**	0.692	0.326	−0.0649	0.115	0.603	0.0375
	**EPS**			**CPS**		
	**Association (** ***p*** **-value)**	**Type (** ***p*** **-value)**	*R*^2^ **value**	**Association (** ***p*** **-value)**	**Type (** ***p*** **-value)**	*R*^2^ **value**
**Observer 1 (*****n*** **=** **97)**	<0.001^***^	0.0208^*^	0.142	<0.001^***^	0.120	0.299
**Observer 2 (*****n*** **=** **94)**	<0.001^***^	0.513	0.133	<0.001^***^	0.571	0.433
**Observer 3 (*****n*** **=** **55)**	0.0479^*^	0.413	0.118	<0.001^***^	0.240	0.298
**Observer 4 (*****n*** **=** **20)**	0.508	0.0992	0.0608	<0.001^***^	0.140	0.493
**Observer 5 (*****n*** **=** **20)**	0.571	0.252	0.0173	0.0418^*^	0.780	0.142

p-values for association between total asymmetry score and total pain score (association), p-values for increase in pain scores before objective gait analysis compared with after (type), and R^2^ values explaining the deviance. Number of pain assessments performed (n) is stated for each observer. Statistical significance is indicated as *p < 0.05,

** p < 0.01,

*** p < 0.001.

**Figure 7:**
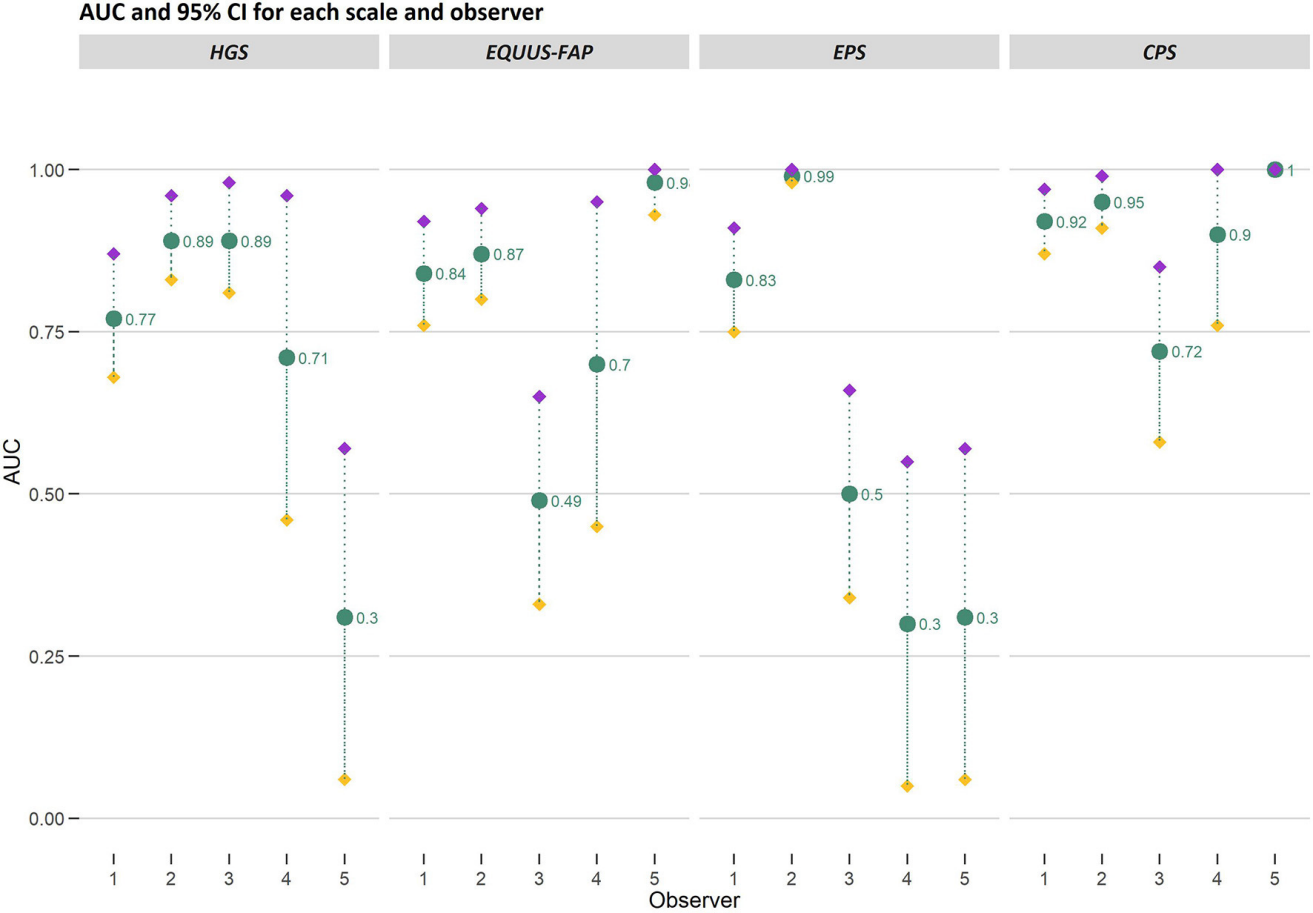
Area under the curve (AUC) and 95% confidence intervals (CI) generated from ROC curves for the Horse Grimace Scale (HGS), Equine Utrecht University Scale of Facial Assessment of Pain (EQUUS-FAP), Equine Pain Scale (EPS), and Composite Orthopedic Pain Scale (CPS). AUC and upper (purple filled diamond) and lower (yellow filled diamond) bounds of CI are presented on the *y*-axis and connected with gray dotted lines. Observer 1–5 are presented on the *x*-axis. The number of observations made by each observer was 97 (observer 1), 94 (observer 2), 55 (observer 3), 20 (observer 4) and 20 (observer 5). The AUC values are stated in the figure.

Area under the curve generated from ROC curves varied among observers and scales (Figure 7). In general, fitted models for observer 1 (*n* = 97 observations) and 2 (*n* = 94 observations) performed better than the models for observer 3, 4 and 5. Based on AUC, observer 1 and 2 could correctly identify horses in pain with HGS with 77%−89% chance, 84%−87% with EQUUS-FAP, 83%−99% with EPS and 92%−95% with CPS. Fitted models for observer 3 (*n* = 55), observer 4 (*n* = 20) and observer 5 (*n* = 20) varied greatly in AUC. For AUC <0.5, it is not possible to distinguish horses in pain from horses without pain, and the random chance is higher. Observer 3 did not succeed in discriminating between “pain” and “no pain” with EQUUS-FAP. Observer 4 did not succeed with EPS, and observer 5 did not succeed with HGS and EPS. Thus was CPS the only scale where all observers succeeded in correctly identifying horses in pain.

## Discussion

Increased movement asymmetry was successfully induced with LPS in all horses – an induction method well-described in horses and known to result in lameness and pain behavior (34–37). This study showed varying performance of four pain scales when assessing low-degree orthopedic pain, but significant linear and non-linear relationships were identified between increases in movement asymmetry and total pain scores given at rest for all scales. Of the four pain scales studied, CPS performed best and pain scores obtained with CPS were most closely associated with movement asymmetry. Progression and regression of movement asymmetry was shown with serial movement asymmetry measurements, beginning 1.5 h post-induction. Lameness progressed and regressed in all horses, as expected from earlier studies (38, 39). Maximum increase in movement asymmetry varied greatly between horses (Figure 1), which is in agreement with previous findings of a highly individual inflammatory response in horses (40) and a wide range in maximum lameness grade (1–4 on an ordinal lameness scale of 0–5) (39). Pain is an experience influenced by external inputs from the surroundings, earlier experience of pain, and compensatory abilities, so individual variance in experienced pain is often present despite standardized pain induction protocols. Use of a within-animal study design where the animals are their own control, as in this study, is therefore recommended (41). To further evaluate the individual pain experience at rest, and since a gold standard for experienced pain is lacking (42), another measure of pain during rest could have been included in our study. Although nociception is different from pain, mechanical nociceptive thresholds in our horses could have been used to demonstrate presence of hyperalgesia around the induced joint as an indicator of inflammatory nociception (38, 43).

In parallel to this, varying degrees of pain behavior were observed at rest, with the majority of pain scores at the low end of each scale's score range. Total pain scores of 0 were sometimes seen post-induction, which may indicate that the horses in our study did not constantly experience pain at rest. Horses are able to decrease the load on the painful limb, resulting in reduced pain intensity and lower pain scores. For instance, facial expressions of pain have been found to be less often present when horses change their posture (8). However, horses with LPS-induced low-grade bilateral orthopedic pain are reported to show no specific behaviors during the presence of lameness (44), and horses with orthopedic disorders may hide their discomfort when observers are present (45). These results indicate that pain can be present despite lack of observed behavioral changes, and that a total pain score of 0 in our study may therefore not be equal to ‘no pain’. In addition, it is often anticipated that the baseline should be zero, which can be misleading when interpreting the magnitude of the scores. In this study, total pain score was higher than 0 before induction on some occasions, especially for EPS. This is an issue rarely discussed in the literature, but positive baseline scores have been described for mice using the Mouse Grimace Scale (46). Further studies are needed to determine baseline intervals and cut-off values for pain in horses.

Despite individual variations, total pain scores, especially those obtained with CPS, were significantly associated with the progression and regression in movement asymmetry, but the asymmetry explained <50% of the variance in pain scores (as illustrated by the *R*^2^ values in Table 2). Based on visual evaluation of the partial effect plots in Figures 3–6, rather high movement asymmetry was present before pain scores increased. In effect, the curve approached a clinically relevant increase in pain score only when TAS reached around 60 mm (see CPS for observers 1–3 in Figure 6). A TAS of 60 mm is a moderate level of lameness, indicating that lower grades of lameness were assigned very low pain scores. Hence, when a resting lameness patient has a total pain score of CPS is >0, the clinician can anticipate that lameness during movement will be present.

When evaluating the AUC as performance parameter, all observers using CPS correctly identified horses in pain with a minimum chance of 72%, which is considered as good performance. This is comparable to the AUC presented for CPS when assessing different types of post-surgical pain in horses using the CPS and Unesp-Botucatu Horse Acute Pain Scale (UHAPS) (47). A difference in performance between observers was present for HGS, EQUUS-FAP and EPS resulting in failure of observer 3–5 to distinguishing between pain and no pain (AUC <0.5). Notably, these observers also had fewer observations than observer 1 and 2, who distinguished between pain and no pain using all scales. This may be interpreted as a need of training to develop skill in using HGS, EQUUS-FAP and EPS before these scales correctly identify pain (48).

Interestingly, both non-linear and linear relationships were seen in the plots in this study, varying between both scales and observers. Hence, an increase in pain score of 20% did not necessarily imply an increase in pain intensity of 20%. Therefore, more research is needed on the clinical meaning of a numerical pain score, especially during pain progression and regression. Furthermore, the relationship between movement asymmetry and LPS-induced pain identified in this study may be very different in horses with chronic lameness, such as osteoarthritis. LPS-induced pain is an acute pain experience not previously encountered by the horse, while most lameness types involve more long-lasting pain experiences where the horse has time to develop a coping behavior. Different degrees of pain may also be present depending on the pain process. For instance, osteoarthritic bone processes may only be painful during motion, whereas LPS-induced synovitis is painful during loading at rest and in motion. This will affect the outcome of pain assessment during rest.

The order of pain assessment and objective gait analysis seemed important for the results obtained using HGS, EQUUS-FAP and EPS. We tested the hypothesis that movement increases pain scores, but found that pain scores were significantly higher before objective gait analysis. This finding may be interpreted in different ways. Movement may decrease joint distension and result in transient pain relief. Alternatively, movement may contribute to concealment of facial or other cues, due to external input, tiredness, or stress during measurements. HGS had significantly higher scores before movement for all observers except observer 5, indicating that pain-related grimaces detected with HGS may decrease or be concealed after movement. If the horses in our study were stressed, there would have been high HGS scores after movement since facial expressions of pain are present in stressed horses experiencing pain (49), and significant increases in HGS scores have been recorded when applying HGS on stressed horses (50). However, the possible influence, especially of stress, on tool performance should be investigated further before pain assessment tools are incorporated into lameness evaluations.

We hypothesized that all scales are highly reliable. We found that the most reliable pain scale was CPS, where the strong agreement between observers is consistent with previous results (5, 7). EPS was moderately reliable, but has not been evaluated previously. The poor and moderate agreement seen for EQUUS-FAP and HGS is inconsistent with previous results showing good or excellent reliability (5, 6). These scales only assess facial expressions, which may affect the reliability since facial configuration seems to be more difficult to appraise than body movements (8). In addition, the more ambiguity there is in descriptions of a category and its scoring, the more training of observers is needed. It may be argued that scales should be designed in such a way that any observer can use them correctly. It has been suggested that before assuming that a pain scale is generalizable, it should be tested with untrained observers unfamiliar with the scale (51). Nonetheless, observers are often trained prior to reliability testing, but standardized training protocols are seldom published (51). The lack of supervised or reference-guided observer training in our study may have impaired the reliability, and evaluation of the reliability on a small set of horses prior to the experiment would perhaps have identified shortcomings in the training. As discussed earlier, especially training prior to using HGS, EQUUS-FAP and EPS might be needed since observers performing fewer pain assessments struggled more often to identify pain than did observers performing more pain assessments. Training on videos and live horses prior to using these scales might improve the reliability and accuracy of identifying pain. However, when comparing the level of observer training in our study with previous studies reporting high reliability for EQUUS-FAP, HGS, and CPS, they did not differ greatly. Observers using EQUUS-FAP familiarized themselves with the scale and trained on horses free from pain prior to reliability testing (5, 52) and observers using HGS had a detailed protocol containing pictures and descriptions during scoring (6, 11). In the study validating CPS (7), no information is given on observer training. The observers in the present study did not train on horses known to be free from pain prior to the experiment, but thoroughly familiarized themselves with the scales and used the same protocol as in the original studies, when available. The observers in previous studies had experience with scoring behaviors and/or horses, and some were veterinary students or veterinarians. This is comparable to the level of experience among observers in the present study (veterinarians, ethologist, and agronomist, all experienced with horses and some with pain scoring). Despite these similarities in observer training and experience, reliability for EQUUS-FAP and HGS was low or moderate in this study, corroborating the claim that re-evaluation of reliability (and validity) may be required when the disease category or the rating conditions are changed (53).

During the controlled circumstances of experimental studies, the presence of affective states of pain could have been documented further by adding certain physiological measures associated with negative valence affects such as pain, for instance heart rate and heart rate variability (54, 55). This is however not feasible during clinical conditions and in order not to disturb the horses more than necessary, such measures were not included in the present study. A limitation in the present study was the small sample size (eight horses), since horses displayed great variation in lameness and intensity of pain – as described in other studies (39). Including more horses might have led to better representation of different pain intensities, but individual variation should not be ignored for the data to be generalizable. A small sample size was selected, primarily due to ethical concerns regarding induction of pain. The association between pain scores at rest and degree of movement asymmetry has not been described previously; complicating sample size calculation prior to the study since the coefficient of determination (*R*^2^) needs to be estimated. In previous studies in which orthopedic pain was induced in the same way as in this study, sample size ranged from 4 to 19, with most studies commonly involving 6–8 horses (7, 34, 36, 37, 39, 40, 56–66). Another limitation was the blinding level of the observers. Knowing that a horse was going to be subjected to induced pain might have resulted in expectation bias, with the observers anticipating that pain would be present and giving higher pain scores (67). The non-blinded observer 1 was the only observer that obtained significantly higher scores with HGS, EQUUS-FAP, and EPS before objective gait analysis. This indicates that seeing the horse move may lead to assigning it a higher pain score before assessment, compared to after. Interestingly, this situation corresponds to that clinical situation where repeated measurements are used, since every pain assessment gives the observer information on the pain status of the horse. The four pain scales were always used in the same order, which may also have induced expectation bias. Since observer 1 was not blinded, the observers were included separately in the statistical models, thereby preventing a potential blinding effect between observer 1 and the other observers from influencing the results. We found comparable results for the non-blinded observer and the blinded observers. However, this is not always the case and the effect of blinding and expectation bias is an important area that should be investigated further.

## Conclusions

We identified significant associations between pain experienced at rest and degree of movement asymmetry for all scales. Pain scores obtained using CPS were most closely associated with movement asymmetry, but movement asymmetry only explained a minor part of the variation in pain scores at rest. Increases in pain score and movement asymmetry did not occur simultaneously and a horse may have rather high movement asymmetry before total pain scores increase. This is an important challenge when assessing orthopedic pain during rest in subtly lame horses, and underlines the relevance of identifying painful orthopedic lesions by other means, for example local or systemic analgesic testing.

All observers managed to distinguish correctly between horses in pain and without pain when using CPS, with excellent accuracy in four out of five observers. However, when using HGS, EQUUS-FAP and EPS some observers were not able to distinguish between horses in pain and without pain. CPS was also the most reliable scale, while low-moderate reliability for the other scales indicate that different pain assessors might assign the equine patient different pain scores despite being familiar with HGS, EQUUS-FAP and EPS.

## Data availability statement

The original contributions presented in the study are included in the article/Supplementary material, further inquiries can be directed to the corresponding author.

## Ethics statement

The animal study was reviewed and approved by Swedish Ethics Committee, diary number 5.8.18-09822/2018.

## Author contributions

KA, PA, EH, and MR designed the study and contributed to the data collection. PA, EH, and MR were responsible for funding acquisition and supervised the project. L-MT and KA were responsible for the statistical analysis and data interpretation. KA prepared the initial draft. All authors made substantial contributions to data interpretation and manuscript revision, and have approved the submitted version.

## Funding

The study was funded by the Swedish Research Council FORMAS (http://www.formas.se/), grant number 2016-01760 (MR). The funders had no role in designing the study, collecting and analyzing data, preparing the manuscript and deciding to publish the results.

## Conflict of interest

The authors declare that the research was conducted in the absence of any commercial or financial relationships that could be construed as a potential conflict of interest.

## Publisher's note

All claims expressed in this article are solely those of the authors and do not necessarily represent those of their affiliated organizations, or those of the publisher, the editors and the reviewers. Any product that may be evaluated in this article, or claim that may be made by its manufacturer, is not guaranteed or endorsed by the publisher.

## Supplementary material

Supplementary File S1 Dataset in an Excel book used for data analysis. Excel book containing two sheets. The sheet “Abbreviations” explains abbreviations used in column headers. The sheet “Dataset” contains symmetry data for all objective gait analyses, and total pain scores for pain assessments before and after the objective gait analyses.
